# Topical Ophthalmic Cyclosporine in the Treatment of Toxic Epidermal Necrolysis

**DOI:** 10.1155/2011/416842

**Published:** 2011-12-27

**Authors:** Zafer Onaran, Gülşah Usta, Mukadder Koçak, Kemal Örnek, Ünase Büyükkoçak

**Affiliations:** ^1^Department of Ophthalmology, Kırıkkale University School of Medicine, Kırıkkale 71100, Turkey; ^2^Department of Dermatology, Kırıkkale University School of Medicine, Kırıkkale 71100, Turkey; ^3^Department of Anesthesiology, Kırıkkale University School of Medicine, Kırıkkale 71100, Turkey

## Abstract

*Aim*. To describe a case of toxic epidermal necrolysis (TEN) with ocular involvement treated with topical ophthalmic cyclosporine. *Case Presentation*. A 20-year-old woman developed TEN following administration of carbamazepine that was prescribed for epilepsy. Ophthalmic examination revealed bilateral pseudomembranous conjunctivitis. She was hospitalized in the intensive care unit and treated with intravenous corticosteroid and immunoglobulin. Topical cyclosporine was used in combination with topical corticosteroids for ocular surface disease. Following two months of ocular treatment, she recovered without any severe ocular complication. Ocular examination at the four-month followup showed a 2 mm of symblepharon in the lower fornix as the sole pathologic finding. *Conclusion*. Topical ophthalmic cyclosporine may contribute to decrease the ophthalmic complications of TEN and should be considered in the acute stage of the disease.

## 1. Introduction

Toxic epidermal necrolysis (TEN) is a rare idiosyncratic mucocutaneous reaction with high mortality rates and is characterized by high fever and widespread blistering exanthema of macules. The incidence is 0.4 to 1.2 cases per million per year in the general population. Destruction and detachment of the epidermis as well as erosions of mucous membranes are the main features of TEN, which is assumed to be induced by drugs. Ocular involvement in TEN includes blisters of the conjunctival surface, and devastating ocular complications such as permanent visual loss are commonly encountered [1].

In efforts to reduce ocular damage, immediate anti-inflammatory and immunosuppressant therapies have been considered due to the immune-related cytotoxic nature of the disease. Topical corticosteroids have been the main agent used for this purpose as well as systemic medication. Nevertheless, ocular sequelae are still problematic in surviving TEN patients.

Herein, we present a case with TEN in whom topical ophthalmic cyclosporine was used, resulting in recovery without any severe ocular complication.

## 2. Case Report

**Figure 1:**
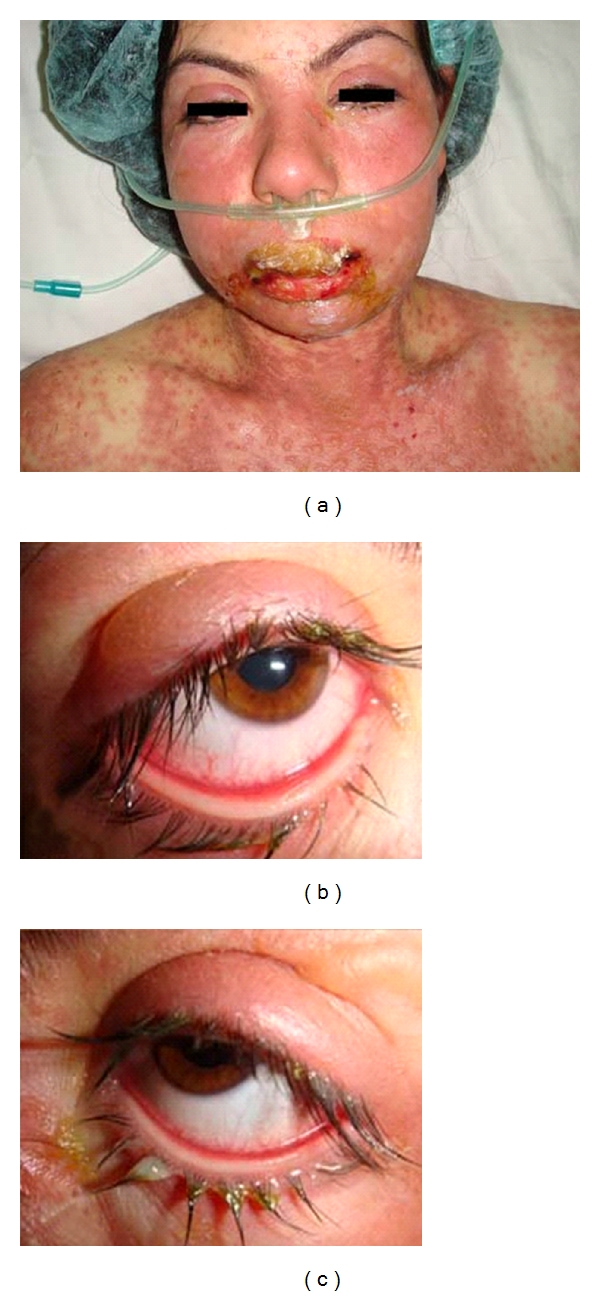
(a) The face and upper trunk of 20-year-old toxic epidermal necrolysis patient manifesting swollen and crusted lips, blisters, and erosions of skin; (b, c) bilateral pseudomembranous conjunctivitis.

A 20-year-old female was admitted to the emergency service with a two-day history of generalized erythematous maculopapular rash. Her medical history was positive for epilepsy. Her neurologist had changed her medication from sodium valproate to carbamazepine 5 days before. Her physical examination revealed widespread red skin with maculopapular and vesiculobullous eruptions and rashes (Figure 1(a)). More than 80% of the patient's total body surface area was involved in addition to the mucous membranes. The patient was tachypneic (130/min), and her body temperature was 38.6°C. The ophthalmic examination revealed vesiculobullous skin lesions of the eyelids. The conjunctiva was hyperemic, and pseudomembranous conjunctivitis was present bilaterally (Figures 1(b) and 1(c)). The cornea and other anterior segment elements were normal.

The clinical diagnosis was consistent with TEN. The patient was hospitalized in the intensive care unit, and carbamazepine was discontinued immediately. Blood and urine cultures were obtained and intravenous teicoplanin (1 × 400 mg) and methylprednisolone (60 mg/day) treatment was initiated the same day. The following day, intravenous immunoglobulin was started, and a total dose of 105 g (2 g/kg) was given in divided doses for 5 days. Sedation was performed using midazolam. Ophthalmic therapy included daily removal of eyelid crusting and topical instillation of preservative-free artificial tears 7 × 1, lomefloxacin 3 × 1, fluorometholone 4 × 1, and ophthalmic cyclosporine 3 × 1.

Disease progression was halted on the 6th day of disease onset, and systemic therapy was terminated on the 8th day. Reepithelization was nearly complete by the 2nd week. She was discharged from the hospital on the 16th day.

**Figure 2:**
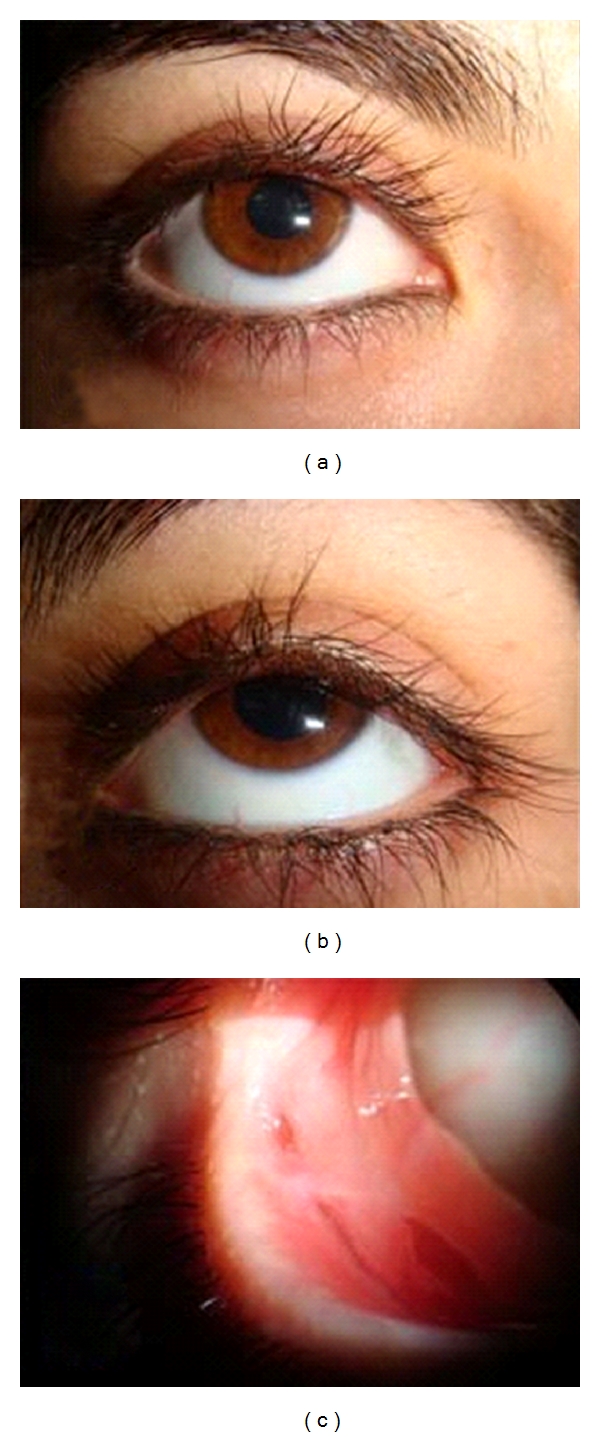
Ophthalmic examination at the fourth month presenting, (a, b) normal anterior segment features, (c) symblepharon formation in the lower fornix of the right eye.

Topical fluorometholone and cyclosporine treatment was continued through the 2nd month. The final ophthalmic examination in the 4th month revealed bilateral visual acuity of 20/20, clear cornea, Schirmer test value >15 mm, and only 2 mm of symblepharon in the lower fornix laterally (Figure 2).

## 3. Discussion

Toxic epidermal necrolysis (TEN) is believed to be an immunologic cytotoxic reaction leading to widespread apoptosis of keratinocytes, with reported mortality rates of approximately 25–35% [2]. As the risk of developing TEN is the highest when the antiepileptic therapy has been recently initiated, and subsequently declines within 8 weeks or more of administration, carbamazepine is supposed to be the causative agent that induced TEN in our demonstrated case where she was on carbamazepine therapy for five days [3].

Genetic susceptibility plays an important role in the pathophysiology of TEN [4]. Immunopathologic studies have demonstrated a clonal expansion of CD8^+^ cytotoxic T lymphocytes, and the production of proinflammatory and costimulatory cytokines apparently plays a central role in TEN. It is now well established that in addition to several apoptotic pathways such as perforin/granzyme or Fas-Fas ligand, secretory granulysin is also involved in the pathogenesis of TEN. Furthermore, regulatory CD4^+^ CD25^+^ T cells have been demonstrated to be potentially important in the prevention of severe epidermal damage by reactive cytotoxic T lymphocytes [5, 6].

Systemic drug therapies have been used to halt this malignant process in order to improve the disease outcome by decreasing mortality rates. These drugs include systemic steroids, thalidomide, intravenous immunoglobulin, cyclophosphamide, tumor necrosis factor-alpha blockers, and cyclosporine. Though some of these agents were reported to have beneficial effects on the disease course, there are no reports indicating a significant effect on reducing ocular complications in frequency or severity [7].

Hence, medical and/or surgical local treatment is mandatory to prevent ocular complications [8]. Conventional treatment includes topical steroids, antibiotics, and tear substitutes. Furthermore, immediate conjunctival adhesiolysis and amniotic membrane grafting are the surgical interventions aimed at reducing ocular morbidity.

In principle, ophthalmic therapy in TEN should primarily focus on suppressing the inflammatory and destructive reaction on the ocular surface. The pathologic process mentioned above seems to be initiated by cytotoxic T-cells. Soluble interleukin-2 receptors (sIL-2Rs), which are considered a marker of T-cell activation, have been detected in high levels in the blister fluid of patients with TEN [9]. This finding addresses the participation of activated T lymphocytes or associated cytokines in the initial steps of the disease.

At this point, cyclosporine could be a good candidate for systemic and topical ophthalmic use as an immunosuppressant agent that acts selectively to suppress T-cell immunity by inhibiting IL-2 expression and preventing proliferation and activation of T lymphocytes.

Recently, in a relatively large group (*n*: 29) of TEN/Stevens-Johnson syndrome (SJS) patients, oral administration of cyclosporine was shown to stop the progression of the disease in the majority of patients and contributed to the survival [10]. However, no data are available about its effect on ocular disease.

Topical ophthalmic cyclosporine has found a large range of indication in inflammatory-/immune-mediated ocular surface diseases, particularly dry eye disease, severe allergic keratoconjunctivitis, and corneal transplant surgery. TEN is on the severe edge of this spectrum, and ophthalmic cyclosporine may be a potential agent in the acute phase management of TEN for limiting the destructive inflammation.

Previously, topical ophthalmic cyclosporine was used by Shammas et al. in the acute stage of SJS and TEN in 8 patients [11]. While they reported preservation of good visual acuity and an intact ocular surface, the role of cyclosporine is not clear, as it had been used in combination with coverage of the entire ocular surface with amniotic membrane.

In conclusion, cyclosporine use in the topical treatment of ocular TEN disease may have beneficial effects in preventing ophthalmic complications. Further investigations need to be conducted with larger series to draw a more definitive conclusion.
